# EBV-associated epithelial cancers cells promote vasculogenic mimicry formation via a secretory cross-talk with the immune microenvironment

**DOI:** 10.7150/thno.100171

**Published:** 2024-08-19

**Authors:** Tong Xiang, Fengze Sun, Tingting Liu, Jingjing Zhao, Jieying Yang, Dijun Ouyang, Hao Chen, Qian Zhu, Qijing Wang, Yongqiang Li, Jia He, Chaopin Yang, Xinyi Yang, Yuanyuan Chen, Yan Tang, Desheng Weng, Qiuzhong Pan, Qi Yang, Jianchuan Xia

**Affiliations:** 1 State Key Laboratory of Oncology in South China, Guangdong Provincial Clinical Research Center for Cancer, Guangdong Key Laboratory of Nasopharyngeal Carcinoma Diagnosis and Therapy, Sun Yat-sen University Cancer Center, Guangzhou, Guangdong, 510060, P. R. China.; 2 Department of Experimental Research, Sun Yat-sen University Cancer Center, Guangzhou, Guangdong, 510060, P. R. China.; 3 Zhuhai People's Hospital (Zhuhai Clinical Medical College of Jinan University).; 4 Department of Medical Imaging, Sun Yat-sen University Cancer Center, Guangzhou 510060, P. R. China.; 5 Department of Biotherapy, Sun Yat-sen University Cancer Center, Guangzhou 510060, P. R. China.; 6 Department of Intensive Care Unit, Sun Yat-sen University Cancer Center, Guangzhou 510060, P. R. China.; 7 Department of Nasopharyngeal Carcinoma, Sun Yat-sen University Cancer Center, Guangzhou 510060, P. R. China.

## Abstract

**Background:** Vasculogenic mimicry (VM) induced by Epstein-Barr virus (EBV) infection plays an important role in resistance to anti-vascular endothelial growth factor (VEGF) therapy in EBV-associated epithelial cancers; however, the interaction between VM and the immune microenvironment has not been systematically investigated.

**Methods:** IHC and multiplex IHC analysis the relationships among tumour-associated macrophage (TAM), VM and EBV infection in EBV-associated epithelial cancer biopsies. *In vitro* and *in vivo* evidence using CRISPR-Cas9 system engineered EBV-infected epithelial cancer cells and mouse models support functional role and mechanism for M2c-like macrophages in the VM formation. The prediction of VM in the effectiveness of anti-angiogenic agent was analysed using clinical datasets.

**Results:** EBV-associated epithelial cancer biopsies revealed that infiltration of the TAM surrounding the VM is closely associated with EBV infection. AKT/mTOR/HIF-1α pathway in EBV-infected epithelial cancer cells control the secretion of CCL5 and CSF-1, enabling the recruitment of monocytes and their differentiation into M2c macrophages which promote VM formation by MMP9. Combination of anti-angiogenesis agents and HIF-1α inhibitor caused marked decreases in CD31-positive micro-vessels, VM, and M2c-like macrophages. VM scores can be used as biomarkers to predict the efficacy of anti-angiogenic agent therapy in EBV-associated epithelial cancers.

**Conclusions:** Our findings define a secretory cross-talk between tumour cells and the immune microenvironment in EBV-associated epithelial cancer, revealing an unexpected role of EBV in epithelial cancer cells, controlling VM formation via M2c-like macrophages.

## Introduction

The Epstein-Barr virus (EBV) was the first human cancer-associated virus to infect more than 90% of the global population 1. Since its isolation from Burkitt's lymphoma 2, EBV has gained considerable attention, primarily due to its oncogenic properties and association with a number of other human malignancies, such as Hodgkin's lymphoma, T cell lymphoma, nasopharyngeal carcinoma (NPC), and EBV-associated gastric carcinoma (EBVaGC) 3. It is estimated that EBV accounts for more than 265,000 cases of cancer each year and that 17% of all cancer deaths are due to EBV-attributable malignancies 4, an increase of 36% in incidence and 19% in mortality in the last two decades 5.

NPC and EBVaGC are the most common EBV-associated epithelial cancers, accounting for 80% of all EBV-associated malignancies 6. Depending on the pattern of EBV gene expression in the infected epithelial cancer cells, two latency programs, referred to as latencies I and II, have been recognised. In most NPC tumours EBV displays type II latency, where EBV-encoded small RNA (EBER), EBV-associated nuclear antigen-1 (EBNA1), latent membrane protein 1/2 (LMP1 and LMP2), and BamHI A rightward transcript (BART)-microRNAs are expressed 7, 8, whereas EBV in EBVaGC has a latency I or II 9, 10. It is believed that the role of EBV in the oncogenic properties of epithelial cancers results from the aberrant establishment of latent viral infections in epithelial cells with premalignant genetic changes 7. Although the mechanism of transformation of premalignant epithelial cells into cancer cells by EBV is uncertain, the presence of EBV in cancer cells has been shown to contribute to the progression of EBV-associated epithelial cancer by promoting cell growth, metastasis, angiogenesis, and resistance to chemotherapy 7. Defining the cellular processes triggered by EBV latency proteins is crucial for understanding the role of EBV in the progression of epithelial cancers and may provide effective therapeutic targets for EBV-associated epithelial cancers.

Vasculogenic mimicry (VM), a new tumour vascular paradigm independent of endothelial cells (ECs), has emerged as another important vasculogenic mechanism in aggressive tumour 11. In a previous study, we reported the role of EBV in promoting VM formation in EBV-associated epithelial cancers through the PI3K/AKT/mTOR/HIF-1α axis by LMP2A, revealing a potential application of HIF-1α as a therapeutic target for EBV-associated epithelial cancers that are resistant to anti-vascular endothelial growth factor (VEGF) therapy 12. However, the interactions between VM and inflammatory cells in EBV-associated cancers have not been systematically investigated. Macrophages, which are largely derived from circulating monocytes, are prominent infiltrative inflammation-related stromal cells present in the tumour microenvironment that display an array of phenotypes, referred to as macrophage polarisation, and are involved in host defence, immune modulation, tissue repair, and tumour neovasculature. Macrophages can be polarised into classically activated (M1) and alternatively activated (M2) cells. Based on different encountered stimuli, M2 macrophages are further divided into M2a, M2b, M2c 13, and M2d subcategories 14. M1-macrophages exhibit pro-inflammatory and anti-cancer functions, while M2-macrophages exhibit immunosuppressive, contribute to matrix remodelling, favouring tumour growth. In this study, we investigated how EBV in epithelial cancers induces the polarisation of tumour-associated macrophages (TAM) to support VM formation through secretion. Thus, we defined a new regulatory role of EBV in epithelial cancer beyond the intrinsic control of VM formation, highlighting the potential clinical utility of macrophage depletion therapeutic strategies for EBV-associated epithelial cancers that are resistant to anti-VEGF therapy. More importantly, VM scores may be used as biomarkers to predict the efficacy of anti-angiogenic agent therapy.

## Materials and methods

**Cell culture.** Akata-EBV-Green fluorescent protein (GFP) is an Burkitt's lymphoma cell line carrying the Akata bacterial artificial chromosome (BAC) with a GFP tag. B95-8 is an EBV-producing marmoset B-cell line that was cultured in RPMI 1640 medium with 10% foetal bovine serum (FBS) (Hyclone). The NPC cell lines HK1 and C666-1 were cultured in RPMI-1640 supplemented with 5% FBS. B and NPC cell lines were obtained from Prof. Mu-Sheng Zeng (Sun Yat-sen University Cancer Center). The gastric carcinoma cell line AGS (ATCC, catalog No. CRL-1739, RRID: CVCL_0139) was kindly provided by Prof. Rui-Hua Xu (Sun Yat-sen University Cancer Center) and cultured in DMEM (Hyclone) containing 5% FBS. THP1 (ATCC, catalog No. TIB-202, RRID: CVCL_0006) was purchased from the ATCC. Patient-derived NPC cells were collected from NPC tissues by nasopharyngeal biopsy after obtaining informed consent from the patient and approval from the Human Research Ethics Committee of the Sun Yat-sen University Cancer Center (GZR2021-029). The patient-derived NPC cells were dissociated into a single cell suspension using a Tumour Dissociation Kit (Miltenyi Biotec, 130-095-929) and a Gentle MACS Octo Dissociator (Miltenyi Biotec). Depletion of non-tumour cells from the dissociated primary tumour tissue was performed by magnetic cell separation using a Tumour Cell Isolation Kit (Miltenyi Biotec,130-108-339), and the separated NPC cells were cultured in RPMI-1640 with 5% FBS. All cells were routinely tested and shown to be mycoplasma-free as determined by a PCR-based method (16S rDNA-F: 5′-ACTCC TACGGGAGGCAGCAGTA-3′, 16S rDNA-R: 5′-TGCACCATCTGTCACTCTGTTAACCTC-3′).

**Clinical specimens.** In total, 10 cases of EBV-positive (seven males and three females, aged 42-61 years, and all with primary NPC tumours) and seven cases of EBV-negative NPCs (five males and two females, aged 35-70 years, and one case of primary tumour and six cases of recurrent tumours), five cases of EBV-positive (five males, aged 33-53 years), and seven cases of EBV-negative human gastric carcinoma (four males and three females, aged 48-84 years) were obtained from the Sun Yat-sen University Cancer Centre, Guangzhou, China, from 2010 to 2017, and all of the samples were diagnosed by two pathologists (Dr. Jinping Yuan and Dr. Jianyong Shao, Sun Yat-sen University Cancer Centre). Ten cases of EBV-positive NPC tumours and one case of primary EBV-negative NPC tumour and human gastric carcinoma did not receive antitumour treatment before biopsy collection; six cases of recurrent EBV-negative NPCs received intensity-modulated radiation therapy with or without platinum-based concurrent chemotherapy.

**Antibodies and chemicals.** The following antibodies were used in this study: Rabbit polyclonal CD68 antibody (ab125212, abcam, 1:200 for IHC), anti-human CD31 antibody (3528, Cell Signaling Technology, 1:100 for IHC), rabbit monoclonal CD163 antibody (ab189915, abcam, 1:200 for IHC), rabbit monoclonal CD206 antibody (ab252921, abcam, 1:2000 for IHC), rabbit monoclonal CD86 antibody (ab269587, abcam, 1:100 for IHC), rabbit monoclonal CCL5 antibody (ab307712, abcam, 1:200 for IHC, 1:1000 for WB), rabbit monoclonal CSF1 antibody (ab206234, abcam, 1:100 for IHC,1:2000 for WB), phospho-AKT (Ser473) (4060, Cell Signaling Technology, 1:100 for IF, 1:1000 for WB), AKT (9272, Cell Signaling Technology, 1:2000 for WB), phospho-mTOR (Ser2448) (2971, Cell Signaling Technology, 1:1000 for WB), mTOR (2983, Cell Signaling Technology, 1:2000 for WB), rabbit monoclonal HIF-1α antibody (ab51608, Abcam, 1:500 for WB), GAPDH (60004-1-1g, Protein-tech, 1:5000 for WB), mouse monoclonal CCR1 (ab233832, abcam, 1:1000 for WB), rabbit monoclonal CCR3 (ab227034, abcam, 1:1000 for WB), rabbit polyclonal CCR5 (ab7346, abcam, 1:1000 for WB), rabbit monoclonal CSF1R (ab221684, abcam, 1:1000 for WB), and horseradish peroxidase (HRP)-conjugated goat anti-mouse/rabbit secondary antibodies, all purchased from Promega. HLA-DR-FITC (Clone L243, eBiosciences, 1:50 for FC), CD86-PE-Cy7 (Clone IT2.2, eBiosciences, 1:20 for FC), CD163-APC (Clone GHI/61, eBiosciences, 1:20 for FC), CD206-PE (Clone 15-2, Biolegend, 1:20 for FC), CD14-PerCP-Cy5.5 (Clone HCD14, Biolegend,1:50 for FACS). Recombinant human CCL5 (300-06-20) CSF1 (300-25-10) were purchased from PeproTech, Recombinant human MMP9(10327-HNAH-20) were purchased from Sino Biological. Endostatin was purchased from Simcere. The CCR5 inhibitor (HY-13004) and the MMP9 inhibitor (HY-135232) were purchased from MCE. The CSF1R inhibitor (S7818), PX-478(S7612), and clodronate liposomes (CP-005-005) were purchased from Liposoma. LY294002(S1105) and Wortmannin(S2758), 2-Methoxyestradiol (2-MeOE2) (S1233) were purchased from Selleck Chem.

**IHC and multiplex IHC.** IHC was performed on formalin-fixed paraffin-embedded (FFPE) sections of clinical NPC and gastric cancer tissues or xenograft mouse tissues, as previously described 12. The tissues were de-paraffinised and rehydrated, and the samples were subjected to citrate-mediated high-temperature antigen retrieval and incubated at 4 °C overnight with the primary antibodies. A semiquantitative scoring was evaluated as follows: 0 for staining in ≤ 1% of cancer cells, 1 for staining in 2-25% of cancer cells, 2 for staining in 26-50% of cancer cells, 3 for staining in 51-75% of cancer cells, and 4 for staining in ≥ 75% of cancer cells. Staining intensity was scored as follows: 0, no staining; 1, weak staining; 2, moderate staining; and 3, strong staining. Two pathologists reviewed the sections. The staining index (0-12) was obtained by multiplying the intensity by the proportion of immunopositive cells of interest. To detect the VM structures, a PAS staining kit (BASO BA-4080A) was used before haematoxylin counterstaining. The VM structure criteria were as follows: (1) vascular-like channels that were lined with EBER+ tumour cells; (2) positive for PAS but negative for CD31 (PAS+/CD31-). Macrophages positive cells were counted in three randomly selected fields. IHC stained slides were reviewed by two pathologists. Multiplex IHC was performed using the PANO 7-plex IHC kit (CAT 0004100100; Panovue). Slides were subjected to antigen retrieval and various cycles of incubation with different primary antibodies, followed by incubation with HRP-conjugated secondary antibodies and TSA. Subsequently, 4′,6-diamidino-2-phenylindole (DAPI) was used to stain nuclei. Images were analysed as previously described 15.

**Transcriptome analysis.** Global gene expression profiles of EBV-positive, EBV-negative, and EBV-genome destruction by CRISPR-Cas9 system cells were studied using RNA-seq analysis, as previously described 12. Gene Ontology (GO) function classification of DEGs was performed using DAVID (https://david.ncifcrf.gov/). A PPI network of DEGs was established using the STRING database (https://string-db.org) 16. Cytoscape software 3.7.2 was used to visualise the DEGs with a minimum interaction score of more than 0.4 17, and we utilised cytoHubba plug-ins to recognise the degree of interaction of hub-gene clustering according to the Maximal Clique Centrality method. In addition, interrelations between the biological networks and DEGs were further analysed using the ClueGO plug-ins of the Cytoscape software 3.7.2 18, with a *p* value less than 0.05 considered as statistically significant.

**ELISA experiments.** The concentrations of CCL5, CSF1, and MMP9 in the CM of tumour cells or supernatants from macrophages were determined using commercially available R&D Systems® ELISA kits. Microwell absorbance was read at 450 nm on a SpectraMax^®^340PC^384^ microplate reader. All samples were quantified based on a standard curve using Microsoft Excel.

**Western blot analysis.** Proteins from different cell lines were extracted with SDS lysis buffer, separated by SDS-PAGE on 10% gels, electrotransferred to PVDF membranes (Millipore), and probed with primary antibodies followed by secondary antibodies, according to standard procedures. Immunoreactive bands were visualised using enhanced chemiluminescence reagents (Pierce) and scanned using a chemiluminescent and fluorescent imaging system (Bio-Rad, United States).

**Human PMBC-derived monocytes isolation.** Peripheral blood mononuclear cells (PBMC) from healthy donors were obtained from the Sun Yat-sen University Cancer Center. PBMC isolation was performed by Lymphoprep™ density gradient separation (07851, Stemcell). Miltenyi MACS was used to isolate the CD14+ monocytes (130-111-549, Miltenyi).

**Migration assay.** The migration assay was carried out using Transwell® chambers (24-well plate format) with a 3.0-μm pore size (Corning, Corning, NY, USA). THP-1- or PMBC-derived monocytes (1 × 10^6^ cells/well) suspended in serum-free medium were seeded in the upper chamber and incubated in the bottom chamber with CM derived from tumour cells. The cells were cultured in a 5% CO2 humidified incubator at 37 °C for 4 or 24 h, fixed in 4% paraformaldehyde for 10 min, and stained with a crystal violet staining solution (Beyotime, Shanghai, China) for 20 min. The cells on the upper side of the filters were removed, and the stained cells on the bottom side were photographed in three independent fields in each well under a light microscope at 100× magnification. The cells were counted using the GraphPad Prism software. All experiments were repeated at least three times.

**Macrophage morphology quantification**. Bright-field images from day six of THP1 or human CD14+ monocytes stimulated with tumour cell CM were used to analyse macrophage morphology using GraphPad Prism software. Data from three independent experiments using cells from healthy donors were analysed.

**Differentiation of PMBC-derived monocytes to macrophages *in vitro*.** For macrophage differentiation, 5× 10^5^ CD14+ monocytes per well (six well dish) were seeded in RPMI1640 medium (GIBCO) and incubated in 5% CO2 at 37 ℃. On day 3, 50% of the medium was replenished with fresh medium containing CM derived from EBV-positive tumour cells, EBV-negative tumour cells, CSF1 receptor inhibitor antibody-treated EBV-positive tumour cells, MMP9 inhibitor-treated EBV-positive tumour cells, and CSF-1 and/or MMP9. The monocytes were then incubated for 3 days. Recombinant human CCL5 (300-06-20) and CSF1 (300-25-10) were purchased from PeproTech. Recombinant human MMP9 (10327-HNAH-20) were purchased from Sino Biological.

**Flow cytometry.** Cells were washed once with FACS buffer, and after FcR blocking (BioLegend), co-stained for antibodies. After 30 min incubation at 4 ℃ in the dark, cells were washed twice with FACS buffer and re-suspended in 500 ml DAPI solution (5 ug/ml, BioLegend) for viability and immediately acquired on a Beckman Coulter CytoFLEX Cytometer and analysed using FlowJo 7.6.5 software. The purity of the isolated CD14+ cells was checked by staining with a CD14 antibody and was routinely > 95% across all experiments.

**Tube formation assay *in vitro*.** The 3D Matrigel tube formation assay was performed as previously described 12. Briefly, 30 μL of Matrigel (BD) was plated in 96-well plates and incubated at 37 °C for 30 min to allow polymerisation. Next, 2 × 10^5^ cells/well were added to the Matrigel layer and grown for 12 h. Three Randomised fields were captured using an inverted microscope and the tubes or junctions were quantified from each image by artificial means.

**Digital spatial profiling.** We selected FFPE slides from two NPC samples (EBV-negative and EBV-positive) and performed spatial transcriptome analysis using the GeoMx DSP. The slides were baked at 60 °C for at least 1 h, de-paraffinised, and rehydrated. The RNA target was exposed by incubating the slides in a proteinase K solution and washed with buffers. The slides were incubated overnight with Cancer Transcriptase Atlas (CTA) probe mix. Cancer cells, M2c like macrophages were identified by fluorescence imaging with antibodies for pan-CK and CD163, respectively. M2c like macrophages were selected based on CD163 positive and pan-CK negative staining within tumour-enriched tissue areas. RNA oligonucleotides from the CTA panel (n = 1812 probes), linked with photocleavable oligonucleotide tags (also referred to as 'barcodes'), were hybridised overnight. The hybridised barcodes were sequentially liberated from each ROI via ultraviolet exposure prior to counting and sequencing on an Illumina HiSeq 2500 platform. FASTQ sequencing files were converted to digital count conversion (DCC) files using NanoString GeoMx NGS Pipeline software. The DCC files were transferred to the GeoMx DSP Data Analysis Suite for quality control and data analysis.

**Animal studies.** For Macrophage depletion by clodronate administration studies, NOD.Cg-Prkdc^em1IDMO^Il2rg^em2IDMO^mice (NOD-Prkdc^null^/L2Ry^null^, NPI) used for the construction of humanized mouse were obtained from BElJING IDMO Co., Ltd. Humanized HLA-A24 transgenic NPI female mice were injected intraperitoneally with clodronate liposomes (150 μl) or PBS liposomes as control. The next day, 5 × 10^6^ HK1 or HK1-EBV cells (HLA-A24) were injected subcutaneously along with 20 ml of clodronate or PBS liposomes into the mice. Next, 150 ml clodronate or PBS liposomes was administered intraperitoneally twice per week. Tumour volume (V) was measured using a caliper every 2 d for the duration of the experiment and calculated using the formula V= (length × width 2)/2. On day 14, mice were culled and tumours were harvested, fixed, and embedded in paraffin using standard protocols (see “IHC and multiplex IHC'' section). Macrophage depletion was confirmed using CD163 IHC staining at the endpoint.

For antitumor drug assessment, when the tumours reached 100-200 mm^3^, the humanized HLA-A24 transgenic NPI female mice were randomised into four groups (n=3 for each group). The control group received the vehicle (0.5% sodium carboxymethyl cellulose (CMC)) daily, and the treatment groups received either endostatin (30 mg kg -1 i.v., twice daily) alone, PX-478 (5 mg kg -1 p.o., every two days) alone, or a combination of endostatin and PX-478. The tumour size was measured every alternate day. At the end of the experiment, the mice were euthanised and the tumours were harvested and weighed.

## Results

### M2c-like macrophages surrounding the VM are closely associated with EBV infection

**Figure 1:**
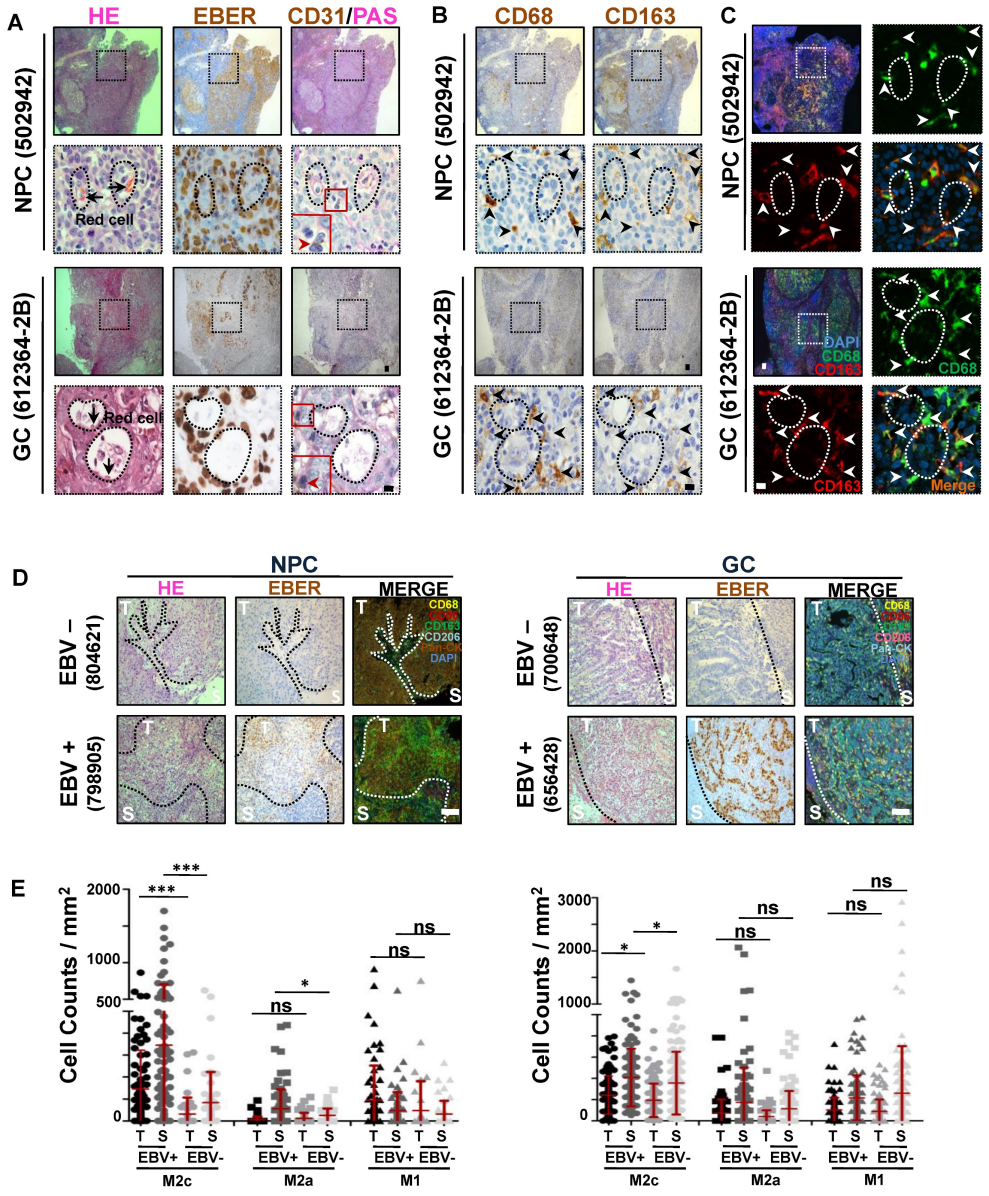
** M2c-like macrophages surrounding the VM are closely associated with EBV infection. A.** (top) NPC and (bottom) EBV-positive gastric carcinoma serial sections were stained with H&E, EBER, PAS, and antibodies targeting human CD31. White scale bars = 100 μm, black scale bars = 10 μm. **B.** (top) NPC and (bottom) EBV-positive gastric carcinoma (GC) serial sections were stained with antibodies targeting human CD68 and CD163. Short black scale bars = 100 μm, long black scale bars = 10 μm.** C.** Immunofluorescence detection of CD68 and CD163-positive cells in NPC tissues. Short white scale bars = 100 μm, long white scale bars = 10 μm. **D.** Multiplex immunohistochemistry analysis of human CD68, CD163, CD206, CD86, and Pan-CK expressions in serial sections of (left) EBV-negative and EBV-positive NPC tissues (left) or EBV-negative and EBV-positive gastric carcinoma (GC) tissues (right). **E.** IHC scores of the indicated macrophage cells in four EBV-negative and seven EBV-positive NPC biopsies (left) or in nine EBV- negative and ten EBV- positive gastric carcinoma biopsies (right). Means ± SD, two-tailed Mann-Whitney test. ns: not significant, *p < 0.05, ***p < 0.001.

We previously reported the role of EBV in promoting VM in EBV-associated tumours 12. Focusing on these VM structures, we found that, in addition to cancer cells, other cell types surrounding the VM primarily showed classical morphological characteristics of macrophages (with a medium-sized oval nucleus or kidney-shaped nucleus) **(Figure 1A, red arrows)**. To further confirm this, we performed immunohistochemical (IHC) staining for the pan-macrophage marker CD68 19, M1-like and M2b-like macrophage markers CD86 20, M2a-like macrophage marker CD206, and M2c-like macrophage marker CD163 21-23 in serial histological sections of EBV-associated tumours. We observed a highly consistent distribution of CD68+ and CD163+ clusters, but not of CD86+ and CD206+ clusters **(Figure 1B and Figure S1A)**. Double-coloured immunofluorescence staining further confirmed CD68 and CD163 co-expression in the infiltrating macrophages around the VM** (Figure 1C)**, showing that the macrophages were indeed M2c-like macrophages. Given that VM in EBV-associated tumours depends on the presence of EBV, we investigated whether EBV was correlated with M2c-like macrophage infiltration. Using the Cancer Genome Atlas (TCGA) database of EBV-positive versus EBV-negative samples of gastric carcinoma 24, we found increased levels of CD163 mRNA in EBV-positive samples **(Figure S1B)**. Furthermore, we conducted tyramide signal amplification (TSA) multiplex immunohistochemical staining for macrophage antibodies in EBV-associated tumour tissues. The analysis, performed using the inForm system, revealed a significant increase in the number of M2c-like macrophages in both the tumour islets and stroma of EBV-positive tissues compared to their EBV-negative counterparts **(Figure 1D-E)**. In order to exclude the interference of gender on the increased number of M2c-like macrophages, we just analyzed the M2c-like macrophages in EBV-associated male tumour tissues. After exclude 3 females NPC patients (1 EBV-positive and 2 EBV-negative) and 3 females GC patients (EBV--negative), we found out that a significant increase in the number of M2c-like macrophages in the EBV-positive male tissues compared to their EBV-negative male counterparts **(Figure S1C)**. To further confirm the EBV was correlated with M2c-like macrophage infiltration in EBV-associated female tumour tissues, we performed IHC staining for M2c-like macrophage in serial histological sections of 5 EBV-positive female GC tissues and 5 EBV-negative female counterparts. The number of M2c-like macrophages in the EBV-positive tissues also increased than that in the EBV-negative female tissues **(Figure S1D-E)**.

Overall, these data indicate that tumour infiltration by M2c-like macrophages surrounding the VM is closely associated with EBV infection.

### EBV-infected epithelial cancer cells favour secretion of macrophage modulatory factors via the AKT/mTOR/HIF-1α pathway

**Figure 2:**
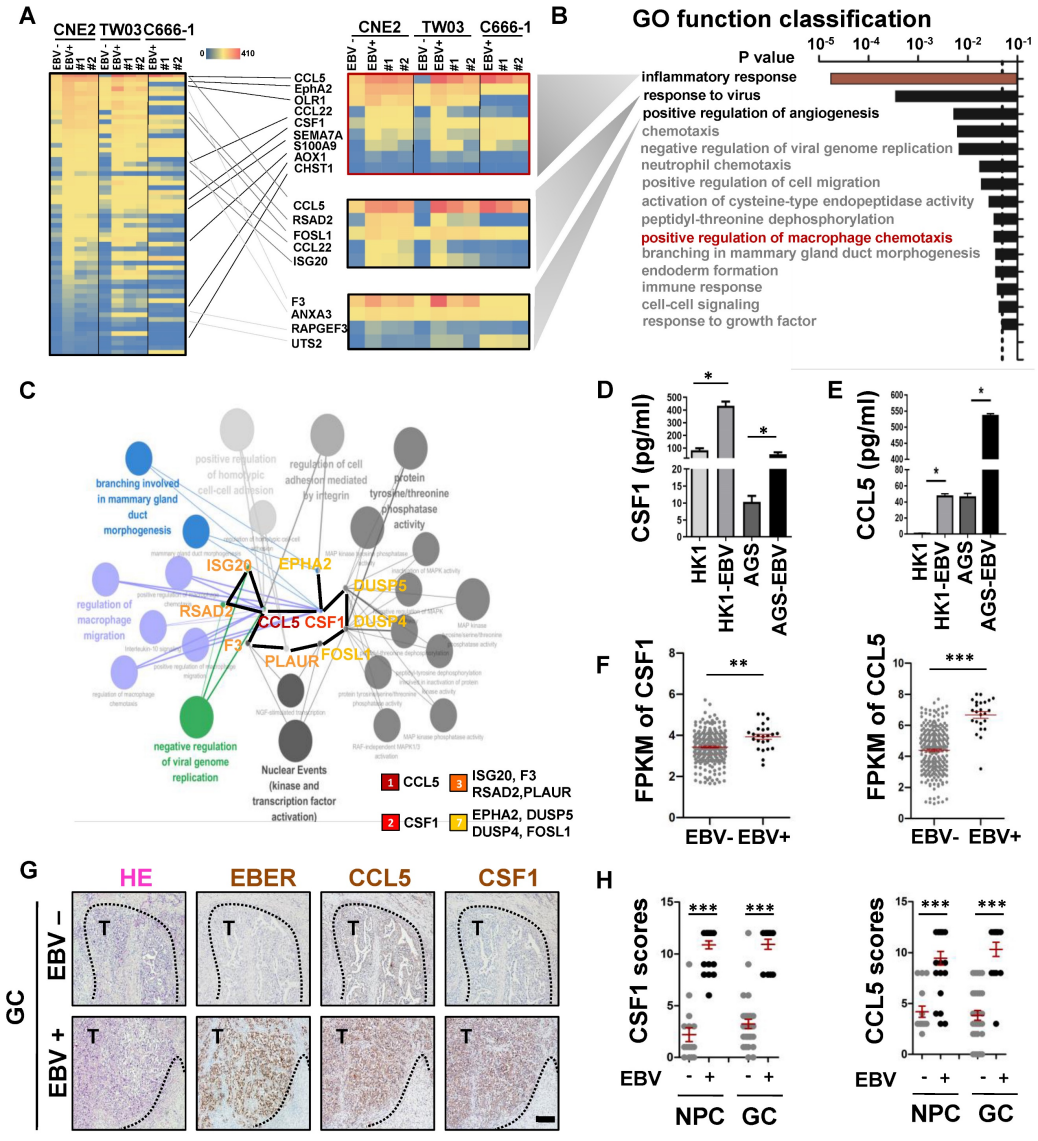
** EBV-infected epithelial cancer cells favour secretion of macrophage modulatory factors via the PI3K/AKT/mTOR/HIF-1α pathway. A.** Heatmap depicting fragments per kilobase of exon per million fragments mapped (FPKM) values for inflammatory response, response to virus and positive regulation of angiogenesis related genes in differentially expressed genes (DEGs) of EBV-negative and EBV-positive NPC cell pairs and EBV-positive cells with EBNA1 deletion by gRNAs. Adjusted (p_adj_) < 0.05. **B.** Bar plot ranking of the top enrichment score (p-value) values for the significant enrichment according to Gene Ontology (GO) function classification of DEGs between EBV-negative and EBV positive NPC cells and EBV positive cells with EBNA1 deletion by gRNAs. Only enrichment with p < 0.05 are shown.** C.** Top 10 candidate core genes from the PPI network of the DEGs (Fig. S2A) were visualised in biological process by Cytoscape with cytoHubba and the ClueGo plug‑in. **D.** ELISA validation for the secreted levels of CSF1 identified in conditioned medium derived from normal epithelial cell lines (N2) and the paired EBV-negative and EBV-positive epithelial cancer cell lines, as indicated. Mean ± SD, n =3, two-tailed Mann-Whitney test. ns: not significant, *p < 0.05.** E.** ELISA validation for the secreted levels of CCL5 identified in conditioned medium derived from normal epithelial cell lines (N2) and the paired EBV-negative and EBV-positive epithelial cancer cell lines, as indicated. Mean ± SD, n = 3, two-tailed Mann-Whitney test. ns: not significant, *p < 0.05.** F.** The FPKM values of the CSF1 and CCL5 in EBV-positive gastric carcinoma cases (n=24) compared to EBV-negative gastric carcinoma cases (n=248). Mean ± SD, two-tailed unpaired t-test. **p < 0.01, ***p < 0.001. **G.** Serial sections of EBV-negative and EBV-positive gastric carcinoma tissues were stained with H&E, EBER, and antibodies targeting CCL5 and CSF1. Scale bars = 500 μm. **H.** IHC scores of the CCL5 and CSF1 in 5 EBV-negative and 8 EBV-positive NPC biopsies or in 10 EBV-negative and five EBV-positive gastric carcinoma biopsies. Mean ± SD, two-tailed unpaired t-test. ***p < 0.001.

It is well known that EBV-infected host cells can communicate with immune microenvironment components via secreted factors 25. Using an RNA-sequencing (RNA-seq) assay, we first identified 60 differentially expressed genes (DEGs) that were consistently upregulated in EBV-infected epithelial cancer cell lines when compared to their EBV-negative counterparts. This observation persisted after modifications to the EBV genome (EBER destruction), achieved through the CRISPR-Cas9 system 12 **(Figure 2A)**. Gene Ontology (GO) function classification using the DAVID tool showed that the DEGs were primarily involved in the inflammatory response, response to viruses, positive regulation of angiogenesis, and positive regulation of macrophage chemotaxis** (Figure 2B)**. To understand the relationships between the proteins encoded by the DEGs, we first used the STRING network tool to construct a protein-protein interaction (PPI) network of the DEGs with 55 nodes and 30 edges **(Figure S2A)**. Cytoscape with ClueGo and the cytoHubba plug‑in were used to visualise the PPI network for these DEGs and screen core genes and biological processes. As observed in **Figure 2C**, the top 10 candidate core genes (*CCL5*, *CSF1*, *ISG20*, *F3*, *RSAD2*, *PLAUR*, *EPHA2*, *DUSP5*, *DUSP4*, and *FOSL1*) were identified and were primarily associated with the regulation of viral genome replication, regulation of macrophages, and branching morphogenesis. Using Enzyme-Linked Immunosorbnent Assay (ELISA), we confirmed that EBV-infected epithelial cancer cell lines secreted high levels of the macrophage modulatory factors CCL5 and CSF-1 **(Figure 2D-E)**, which were the most significant terms among the 10 candidate core genes. To expand our observations to the clinical setting, TCGA databases (NPC 26 and GC 24) were used to evaluate mRNA levels of the highly secreted macrophage modulatory factors by EBV-infected epithelial cancer cell lines. CCL5 and CSF1 mRNA levels were upregulated in EBV-positive epithelial cancer samples compared to those in EBV-negative samples **(Figure 2F),** suggesting transcriptional regulation. The upregulation of CCL5 and CSF1 was further confirmed by IHC staining in EBV-positive epithelial cancer samples compared to EBV-negative samples **(Figure 2G-H, Figure S2B)**. The AKT/mTOR/HIF-1α signalling cascade is a key axis in VM formation of EBV-infected epithelial malignancies 12. As expected, EBV infection led to an increase phosphorylation of AKT/mTOR/HIF-1α as well as the expression of CCL5 and CSF1. In addition, two PI3K inhibitors (LY294002 and Wortmanin) and two HIF-1α inhibitors (2MeOE2 and PX-478) markedly reduced the phosphorylation of AKT/mTOR as well as the protein levels of HIF-1α, CCL5, and CSF1 (**Figure S2C**). EBV typically exists in a latent form, and the latent gene LMP2A is consistently detected in various EBV-associated epithelial malignancies. Over-expression of LMP2A in EBV-non infected epithelial cancer cells (HK1 and AGS) led to increases levels of phosphorylation of AKT/mTOR, accompanied by upregulation of HIF-1α, CCL5, and CSF1. Moreover, knockdown of LMP2A with siRNA reduced AKT/mTOR signalling activation and HIF-1α, CCL5, and CSF1 expression (**Figure S2D**).

Overall, our data show that the macrophage modulatory factors CCL5 and CSF1, which are important in EBV-infected epithelial malignancies progression, are regulated by AKT/mTOR/HIF-1α pathway axis.

### EBV-associated epithelial cancer cells increase monocyte migration

**Figure 3:**
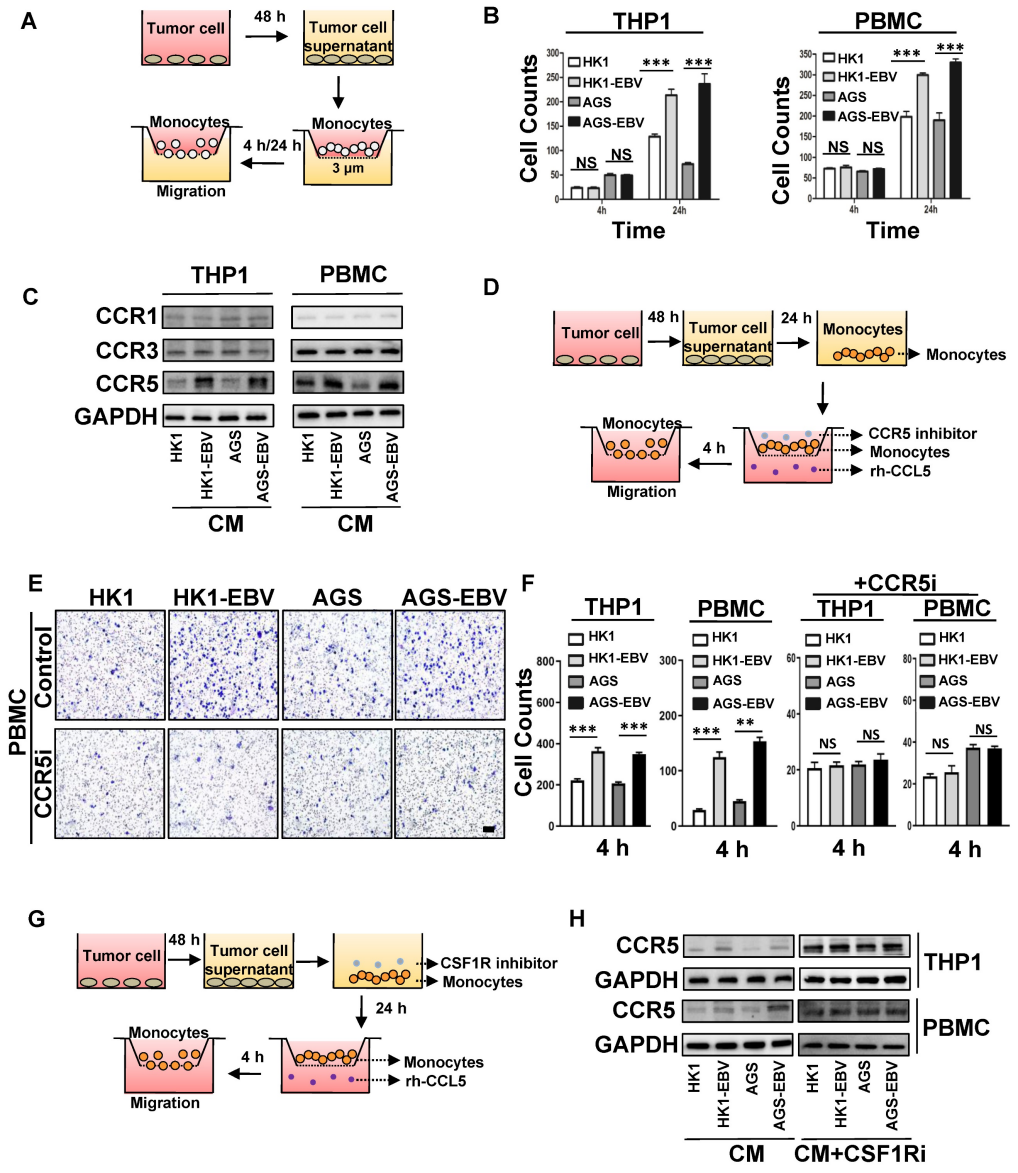
** EBV-associated epithelial cancer cells increase monocyte migration. A.** Schematic: *in vitro* treatment of monocytes with conditioned media CM from tumour cells and subsequent migration assay. **B.** Quantification of monocyte migration induced by CM of EBV-infected epithelial cancer cells and their parental cells. Cells were counted under a microscope with 200× magnification in randomly chosen fields. Mean ± SD, n = 3, ns: not significant, ***p < 0.001 (two-tailed unpaired t-test).** C.** Levels of CCR1, CCR3, and CCR5 on the THP1 and PBMC-derived monocyte stimulated by EBV-positive tumour cells CM after 24 h.** D.** Schematic:* in vitro* treatment of monocytes with conditioned media CM from tumour cells and subsequent migration assay in the presence of CCR5 inhibitor and rh-CCL5.** E.** Images of monocyte migration induced by CM of EBV-infected epithelial cancer cells and their parental cells in the presence of CCR5 inhibitor and rh-CCL5. Images were taken 4 h after seeding on chamber. Scale bars = 100 μm. **F.** Quantification of monocyte migration induced by CM of EBV-infected epithelial cancer cells and their parental cells in the presence of CCR5 inhibitor and rh-CCL5. Cells were counted under a microscope with 1000× magnification in randomly chosen fields. Mean ± SD, n = 3, ns: not significant, **p < 0.05, ***p < 0.001 (two-tailed unpaired t-test). **G.** Schematic: *in vitro* treatment of monocytes with conditioned media CM from tumour cells in the presence of CSF1R inhibitor for 24 h and subsequent migration assay in the presence of rh-CCL5 for 4 h.** H.** CCR5 levels on the THP1 and PBMC-derived monocyte stimulated by EBV-positive tumour cells CM and CM+CSF1R inhibitor after 24 h.

Tumour infiltration of M2c-like macrophages surrounding the VM in EBV-infected epithelial cancer **(Figure 1)** suggests potential communication between cancer cells and macrophages. On the other hand, the DEGs in EBV-infected epithelial cancer cells were rich in macrophage-modulatory factors **(Figure 2C)**. Next, we investigated the interaction between M2c-like macrophages and EBV-associated tumour cells *in vitro* using a transwell co-culture system. First, we investigated the role of EBV-associated tumour cells in the regulation of monocyte chemotaxis, which is a macrophage precursor **(Figure 3A)**. We hypothesised that EBV-associated tumour cells would regulate the migration of monocytes towards endogenous macrophage modulatory factors expressed in their conditioned media (CM). The migration of monocytic cell lines (THP-1) and human peripheral blood mononuclear cell (PMBC)-derived monocytes did not increase towards the CM derived from EBV-positive tumour cells versus EBV-negative tumour cells in 4 h **(Figure 3B and Figure S3A)**. The timescale of this assay (2-4 h) 27 suggests that increased macrophage modulatory factors in the CM of EBV-associated tumour cells do not exhibit direct chemokinetic properties in monocytes. Interestingly, the migration of THP-1- and PMBC-derived monocytes increased towards CM derived from EBV-positive tumour cells versus EBV-negative tumour cells after 24 h **(Figure 3B and Figure S3B)**. This suggests that the enhancement of chemotaxis may stem from receptor regulation of macrophage modulatory factors. CM-stimulated monocytes were further investigated for the surface expression of macrophage modulatory factor receptors. Over the course of 24 h, EBV-positive tumour cell CM-stimulated monocytes had no significant effect on the surface expression of CSF1 receptor (CSF1R) compared to EBV-negative tumour cells **(Figure S3C)**; however, a significant increase in CCR5 expression was observed on the monocyte surface **(Figure 3C)**. These results demonstrate the regulation of CCR5 surface expression by monocytes which correlates with the regulation of CM-induced migration.

The promotion of monocyte CCR5 surface expression by EBV-positive tumour cell CM correlated with an enhancement of the macrophage modulatory factor towards CCR5-chemokines, CCL5 **(Figure 2E)**. To investigate whether CCR5 expression was responsible for the promotion of migration towards CCL5, we repeated chemotaxis experiments in recombinant human CCL5 (rh-CCL5) or CCR5-inhibited system **(Figure 3D)**. rh-CCL5 significantly promoted the migration of THP-1 and PMBC-derived monocytes in 4 h, which was stimulated by EBV-positive tumour cell CM for 24 h. Treatment of stimulated THP-1 and PMBC-derived monocytes with a CCR5 blocking antibody partially decreased the migration towards rh-CCL5 **(Figure 3E-F, Figure S3D)**. These data suggest that EBV-positive tumour cell CM can promote CCR5 receptor expression on monocytes, and that CCL5 secreted by tumour cells is involved in the migration of monocytes towards tumour cells. Previous studies have reported that CSF-1 can upregulate the expression CCR5 in monocytes of patients infected with HCV 28. Similarly, treatment with recombinant human CSF1 (rh-CSF1) significantly increased the expression CCR5 in monocytes after 24 h **(Figure S3E)**. The migration of THP-1- and PMBC-derived monocytes stimulated with rh-CSF1 for 24 h was significantly promoted towards rh-CCL5 at 4 h **(Figure S3F)**. To investigate whether the upregulation of CSF-1 in EBV-associated tumour cells regulates CCR5 receptor expression on monocytes and migration towards rh-CCL5, we performed chemotaxis experiments in an rh-CCL5 and CSF1 receptor (CSF1R)-inhibited system **(Figure 3G)**. EBV-positive tumour cell CM-stimulated monocytes with a CSF1 receptor (CSF1R)-neutralising antibody had no significant effect on the surface expression of CCR5 **(Figure 3H)**.

Overall, these data suggest that increased expression of CSF-1 in EBV-associated tumour cell CM can upregulate CCR5 receptor expression on monocytes and migration towards increased CCL5 in EBV-associated tumour cell CM.

### EBV-associated epithelial cancer cells induce M2c-like macrophages differentiation

**Figure 4:**
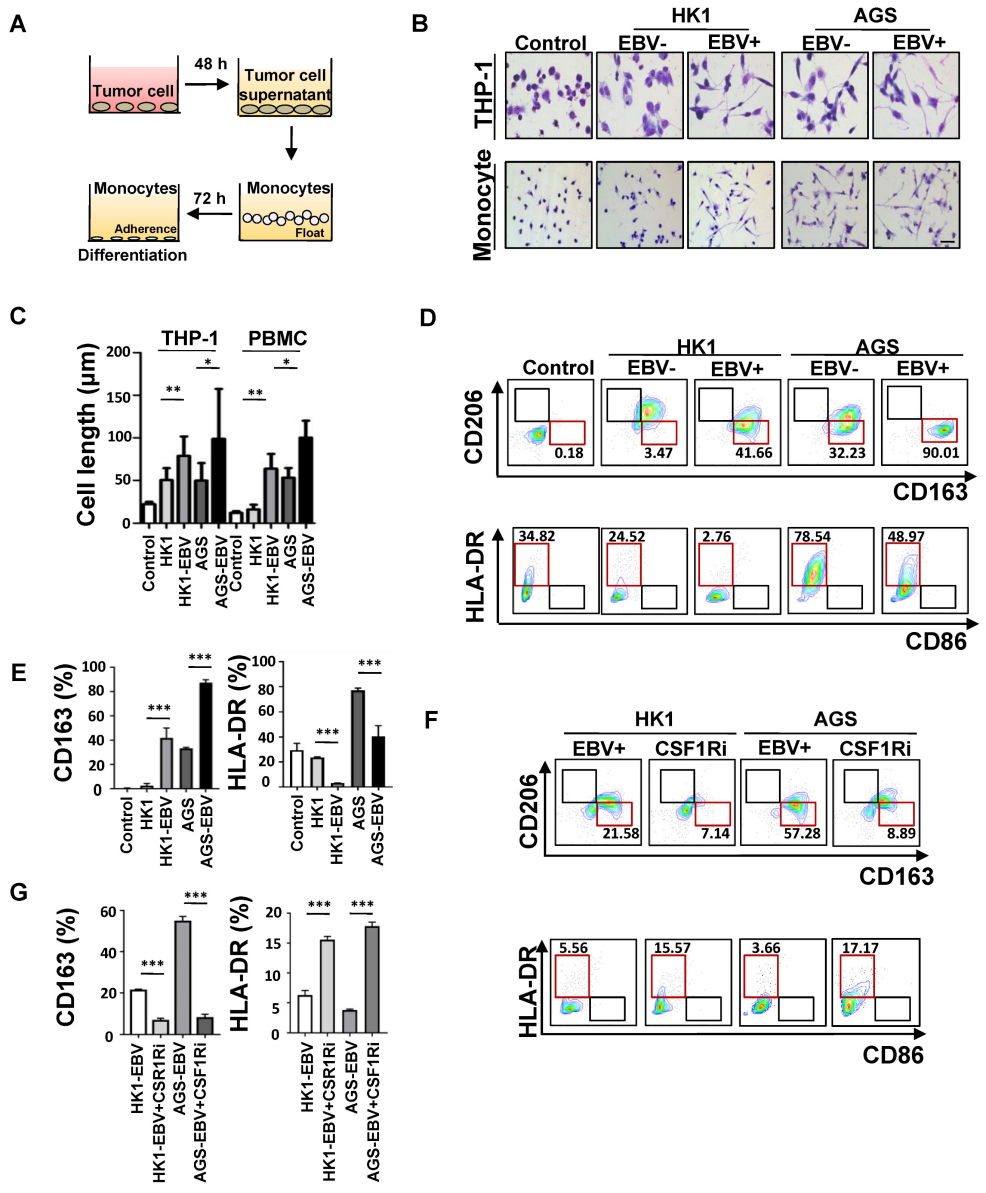
** EBV-associated epithelial cancer cells induce M2c-like macrophages differentiation. A.** Schematic: *in vitro* treatment of monocytes with conditioned media (CM) from tumour cells and subsequent differentiation assay.** B.** Image of macrophage morphology induced by CM of EBV-infected epithelial cancer cells and their parental cells. Images were taken 72 h after seeding on chamber. Scale bars = 50 μm. **C.** Quantification of macrophage cell length induced by CM of EBV-infected epithelial cancer cells and their parental cells. Mean ± SD, two-tailed unpaired t-test. *p < 0.05, **p < 0.01.** D.** Flow cytometry dot plots from PMBC-derived monocytes of one donor showing CD163, CD206 HLA-DR, and CD86 levels after treatment with CM of EBV-infected epithelial cancer cells and their parental cells or culture media only (control).** E.** %CD163 and %HLA-DR levels after treatment with CM of EBV-infected epithelial cancer cells and their parental cells, or culture media only (control) (n = 3; 3 different healthy donors). Mean ± SD, two-tailed unpaired t-test. ***p < 0.001. **F.** Flow cytometry dot plots from PMBC-derived monocytes of one donor showing CD163, CD206 HLA-DR, and CD86 levels after treatment with CM of EBV-infected epithelial cancer cells and CM of EBV-infected epithelial cancer cells in the presence of CCR5 inhibitor. **G.** %CD163 and %HLA-DR levels after treatment with CM of EBV-infected epithelial cancer cells and CM of EBV-infected epithelial cancer cells in the presence of CSF1R inhibitor (n=3; 3 different healthy donors). Mean ± SD, two-tailed unpaired t-test. ***p < 0.001, ns: not significant.

Next, we addressed whether monocytes could differentiate into M2c-like macrophages in response to CM derived from EBV-positive tumour cells versus EBV-negative tumour cells **(Figure 4A)**. Cell elongation, a characteristic of M2-like macrophage 29, was induced more efficiently in the CM of EBV-positive tumour cell-treated monocytes, but minimally in EBV-negative tumour cell-treated monocytes (**Figure 4B-C**). CD163 expression was increased, and the classic activation marker HLA-DR was decreased in monocytes induced by the CM of EBV-positive tumour cells compared with EBV-negative tumour cells (**Figure 4D-E**). To investigate whether the upregulation of CSF-1 in EBV-associated tumour cells would regulate CD163 expression on monocytes, PMBC-derived monocytes were stimulated by EBV-positive tumour cell CM with CSF1 receptor inhibitor antibody. We found that CD163 expression level was decreased and HLA-DR was increased (**Figure 4F-G**). We further used CM derived from patients with EBV-positive and EBV-negative malignancies to treat paired PMBC-derived monocytes. CM of patients with EBV-positive malignancies induced higher levels of CD163+CD206- macrophages compared to that of patients with EBV-negative malignancies **(Figure S4A -B)**. These data indicated that the CM of EBV-associated tumour cells can induce an M2c-like phenotype in human monocyte-derived macrophages.

### M2c-like macrophages promote VM formation

**Figure 5:**
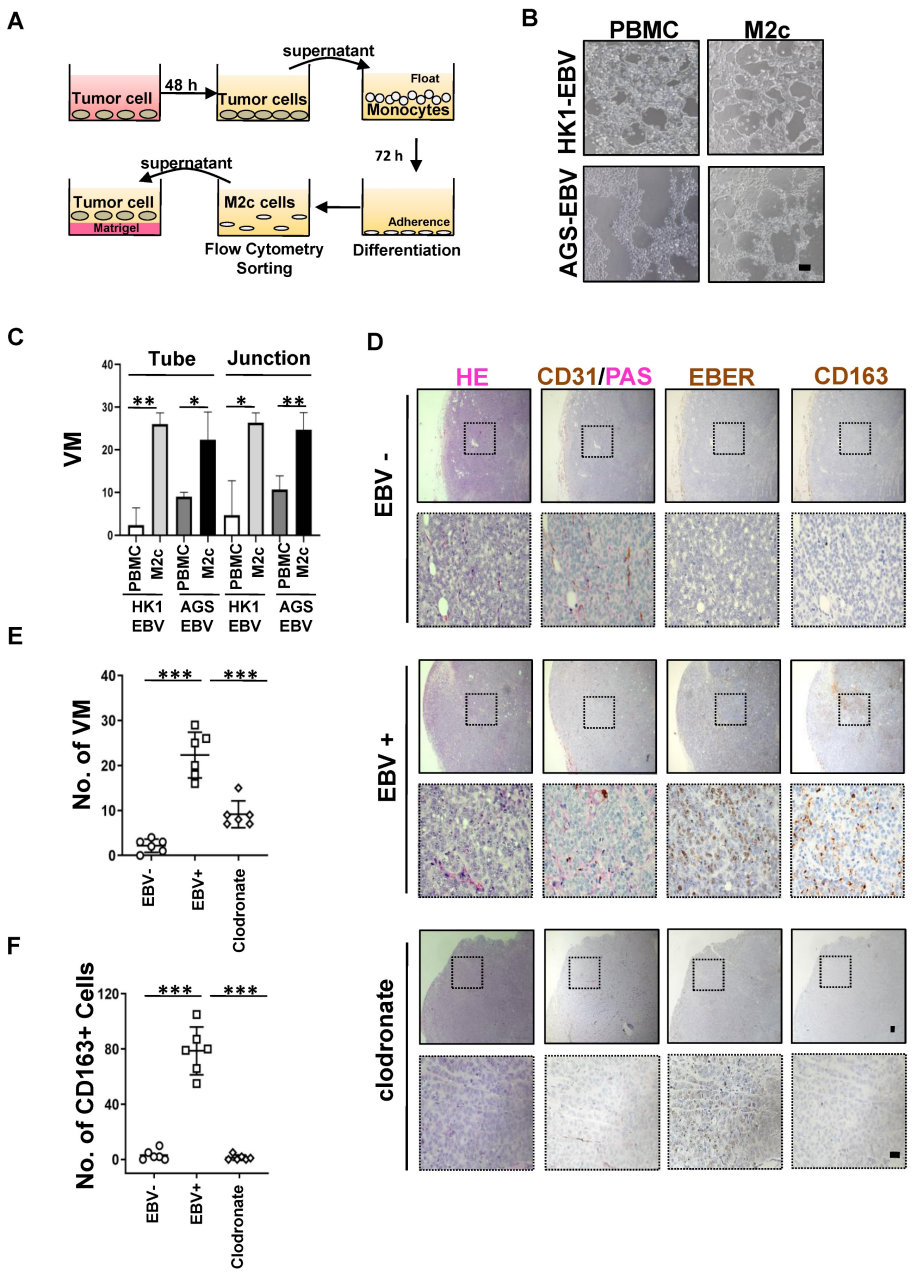
** M2c-like macrophages promote VM formation. A.** Schematic: *in vitro* treatment of PBMC-derived monocytes with conditioned media from tumour cells and subsequent VM formation assay. **B**. Light microscopy of the tube formation of HK-EBV and AGS-EBV cells that treated with supernatant of M2c-like macrophage on Matrigel for additional 12 h. Scale bars = 100 μm. **C**. Quantification of the tube numbers and junction numbers of VM formed by HK-EBV and AGS-EBV cells that treated with supernatant of M2c-like macrophage on Matrigel for additional 12 h. Mean ± SD, n=3, two-tailed unpaired t-test, *p < 0.05, **p < 0.01.** D**. H&E, PAS mouse CD31 staining, EBER, and CD163 of HK1, HK1-EBV, and HK1-EBV treated macrophage depletion clodronate liposome xenograft sections. Short black scale bars = 100 μm, long black scale bars = 10 μm.** E**. Number of VM channel-like structures counted as in D. Mean ± SD, n = 6, two-tailed unpaired t-test, ***p < 0.001. **F**. Number of CD163 positive cells counted as in D. Mean ± SD, n=6, two-tailed unpaired t-test, ***p < 0.001.

We characterised the functional role of M2c-like macrophages induced by EBV-associated tumour cells in the VM. A 3D Matrigel tube formation assay was performed using M2c-like macrophages (CD163+ cells) which were sorted by flow cytometry **(Figure 5A)**, as M2c-like macrophages can promote the formation of VM on Matrigel **(Figure 5B-C)**. Because mouse CCR5 differs significantly from human CCR5 30, it cannot function as a chemoattractant receptor for CCL5; thus, xenograft tumour model was insufficient to permit the entry of monocytes into the tumour for differentiation to TAM. Therefore, to further validate the VM-promoting role of M2c-like macrophages induced by EBV-positive tumour cells *in vivo*, we evaluated the interaction between VM and macrophages in an improved humanised mouse model **(Figure S5A)**. The number of M2c-like macrophages surrounding the VM was also significantly increased in EBV-positive tissues compared to their EBV-negative counterparts **(Figure 5D-E).** Macrophage depletion after clodronate liposome delivery 31 resulted in impaired VM *in vivo* in EBV-positive tumour cells in a humanised xenograft mouse model **(Figure 5D-F)**. These data suggest a key role for M2c-like macrophages in mediating VM in EBV-positive tumours.

### MMP9 mediated the M2c-like macrophages to promote VM

To explore the potential molecular mechanisms by which M2c-like macrophages promote VM, we applied GeoMx Digital Spatial Profiler (DSP) technology for the quantitative analysis of differentially M2c-like macrophage-expressed genes between EBV-positive and EBV-negative samples. Formalin-fixed paraffin-embedded (FFPE) tissue sections were stained with fluorescence-labelled primary antibodies targeting CD163 for M2c-like macrophages and PanCK for tumours.

**Figure 6:**
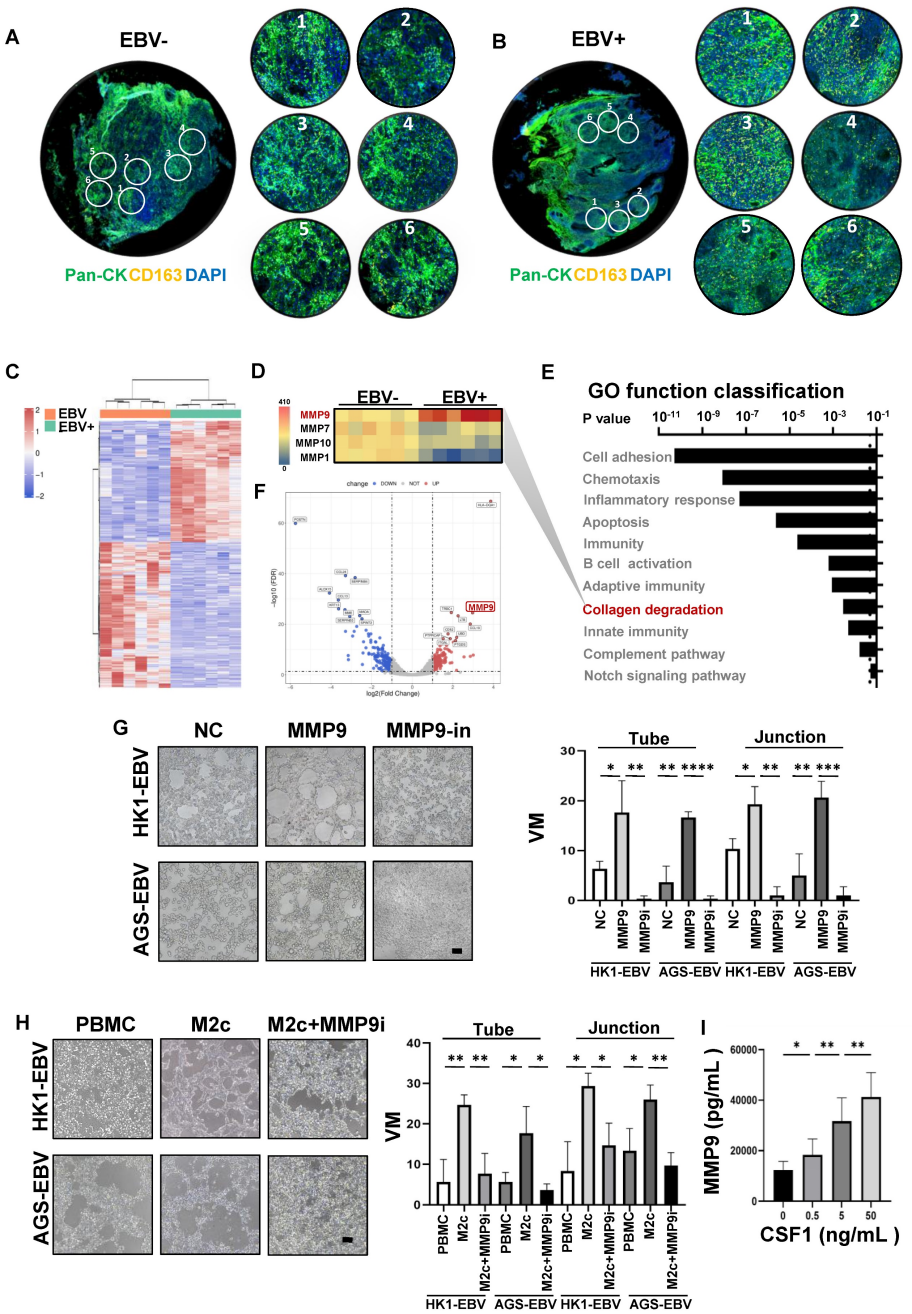
** MMP9 mediated the M2c-like macrophages induce VM formation. A.** EBV-negative NPC tissue sections were stained with morphological markers (epithelial cell marker PanCK, macrophage marker CD163, and nuclear staining with DAPI) to select ROIs. On average, six ROIs were chosen for each section based on staining patterns and spatial features.** B.** EBV-positive NPC tissue sections were stained with morphological markers (epithelial cell marker PanCK, macrophage marker CD163, and nuclear staining with DAPI) to select ROIs. On average, six ROIs were chosen for each section based on staining patterns and spatial features. **C.** Heatmap depicting FPKM values for collagen degradation related genes in EBV-negative and EBV-positive NPC cell pairs. p_adj_ < 0.05. **D.** Heatmap depicting FPKM values of differentially expressed genes in EBV-negative and EBV-positive NPC cell pairs. p_adj_ < 0.05. **E.** Bar plot ranking of the top enrichment score (p-value) values for the significant enrichment pathways according to GO function classification analysis of differentially expressed genes between EBV-positive and EBV-negative NPC cells. Only pathways with p < 0.05 are shown. **F.** Volcano plot for differentially expressed genes between EBV-positive and EBV-negative tissues. FDR<0.05 and Log2(Fold Change)>1. **G.** Representative images (left) and quantification (right) of *in vitro* treatment of MMP9 and its inhibitor (MMP9i) with conditioned media (CM) from tumour cells and subsequent VM formation assay. Scale bars = 100 μm. Mean ± SD, two-tailed unpaired t-test. *p < 0.05, **p < 0.01, ***p < 0.001, ****p < 0.0001.** H.** Representative images (left) and quantification (right) of *in vitro* treatment of M2c-macrophages and MMP9 inhibitor (MMP9i) with conditioned media (CM) from tumour cells and subsequent VM formation assay. Scale bars = 100 μm. Means ± SD, two-tailed unpaired t-test. ***p < 0.001.** I.** MMP9 secretory protein levels after CSF1-treated in M2c-like macrophage. Means ± SD, two-tailed unpaired t-test. *p < 0.05, **p < 0.01.

RNA-seq for M2c-like macrophages was performed on 12 regions of interest (ROIs) **(Figure 6A-C)** and analysed by edgeR and DAVID tool between EBV-negative and EBV-positive samples. We observed high MMP9 expression involved in the collagen degradation, which plays an important role in VM 32 **(Figure 6D-F)**. To further validate the VM-promoting role of MMP9, we performed an in vitro treatment of MMP9 on EBV-positive tumour cells and subsequent VM formation assays. MMP9 promotes the formation of VM on Matrigel; however, VM formation is blocked by an MMP9 inhibitor **(Figure 6G)**. We also performed *in vitro* treatment with M2c-macrophages and MMP9 inhibitors on EBV-positive tumour cells and subsequent VM formation assays. M2c-macrophage was found to increase the VM formed on Matrigel, whereas the MMP9 inhibitor halted this process **(Figure 6H)**. Since CSF1 and MMP9 have been found to promote M2c-like macrophage differentiation and VM formation, we investigated whether CSF1 could induce MMP9 expression in monocytes. Our data showed that MMP9 secretory protein levels were significantly higher in CSF1-treated M2c-like macrophages **(Figure 6I).**

Collectively, these findings indicated that VM in EBV-related tumour cells can be promoted by the secretion of MMP9 from M2c-like macrophages.

### Synergistic effect of anti-VM and anti-VEGF therapy in EBV-associated epithelial cancer

**Figure 7:**
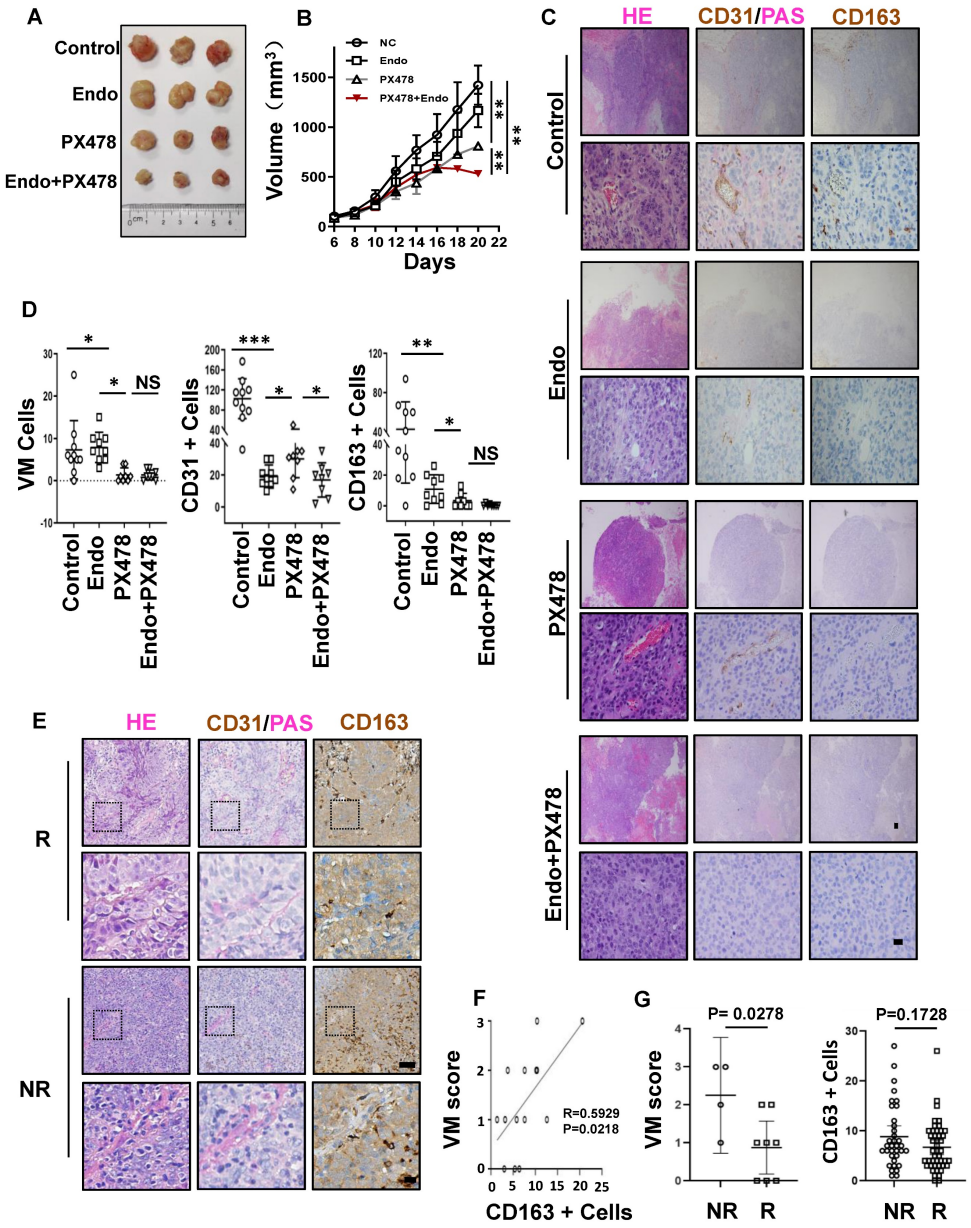
** Synergistic effect of anti-VM and anti-VEGF therapy in EBV-associated epithelial cancer. A.** Image of HK1-EBV xenograft tumours from mice treated with control, endostatin (30 mg kg -1 i.v., twice daily) alone, PX-478 (5 mg kg -1 p.o., every two days) alone, or a combination of endostatin and PX-478. **B.** Growth curves of HK1-EBV xenograft tumours after treatment with vehicle, endostatin and/or PX-478. Mean ± SD, two-tailed unpaired t-test, **p < 0.01. **C.** H&E, PAS, CD31, and CD163 staining of HK1-EBV xenograft sections. Short black scale bars = 100 μm, long black scale bars = 10 μm. **D.** Number of VM cells, CD31-positive cells, and CD163-positive cells in HK1-EBV xenograft tumours from mice treated with the vehicle, endostatin and/or PX-478. Numbers were determined under a microscope with 400× magnification in randomly chosen fields. Mean ± SD, n = 10, two-tailed unpaired t-test. *p < 0.05, **p < 0.01, ***p < 0.001. **E.** H&E, PAS and CD31 staining, CD163 staining in patients treated with endostatin. long scale bars = 50 μm, short scale bars = 10 μm. **F.** Relevance of VM scores and M2c-like macrophages. Correlation coefficients were calculated by Spearman correlation analyses. Each dot represented one sample. **G.** Relevance of response and VM scores (left) or CD163-positive Cells (right). Correlation coefficients were calculated by Spearman correlation analyses. Each dot represented one sample. NR: no response including stable diseases (SD) and progression disease (PD) patients, R: response patients including complete response (CR) and partial responses (PR) patients.

Anti-angiogenic agents that target the classic angiogenic pathway (such as VEGF or ECs) have already been used for the treatment of EBV-associated epithelial cancers. Recently, in a phase-2 trial, endostatin evidenced promising efficacy in patients with metastatic NPC in chemotherapy 33. Mechanistically, however, endostatin did not have an appreciable effect on VM in NPC 34. Thus, we propose that the concurrent targeting of classic angiogenesis and VM may achieve maximal antitumour efficacy in EBV-associated epithelial cancers. Moreover, we observed that VM formation in EBV-associated epithelial cancers dependent on the HIF-1α mediated crosstalk between cancer cells and M2c-like macrophages. We further evaluated the effects of anti-VEGF monotherapy, anti-HIF-1α monotherapy, and combination therapy in NPC xenografts. Treatment with the HIF-1α inhibitor PX-478 or the VEGFR inhibitor endostatin alone only showed modest antitumour effects. In contrast, the combination of PX-478 and endostatin induced a significantly more pronounced tumour inhibition **(Figure 7A-B)**. We assessed tumour neovascularization using histological double staining for CD31 and Periodic Acid-Schiff stain (PAS). The tumours in the control group were enriched with ECs, VM channel-like structures, and M2c-like macrophages. Endostatin decreased CD31 positive micro-vessels without affecting VM, whereas PX-478 treatment led to large areas of VM and M2c-like macrophage loss without a significant reduction in CD31-positive micro-vessels. Notably, the combination of endostatin and PX-478 markedly decreases of CD31-positive micro-vessels, VM, and M2c-like macrophages** (Figure 7C-D)**. Subsequently, we explored predictive biomarkers for patients treated with endostatins. Patients who responded to endostatin treatment were characterised by VM scores and M2c-like macrophages **(Figure 7E)**. There was a strong correlation between VM scores and M2c-like macrophages **(Figure 7F)**. In addition, a high VM score was associated with a worse objective response rate of patients **(Figure 7G)**. These results suggest that targeting VEGF and HIF-1α has synergistic anti-angiogenic effects, providing a novel therapeutic option for EBV-associated tumours. VM scores can be used as biomarkers to predict the efficacy of anti-angiogenic agent therapy.

## Discussion

Anti-angiogenic therapy has demonstrated success in certain solid tumours. However, the use of anti-angiogenic therapies results in a hypoxic tumour microenvironment and the development of resistance by alternative tumour vascularization 35. Accumulating evidence suggests that VM may be a promising therapeutic strategy to overcome the limitations of anti-angiogenic therapy in patients with cancer 36. We previously reported a role for EBV in promoting VM in EBV-associated epithelial cancers through the PI3K/AKT/mTOR/HIF-la axis by LMP2A and demonstrated its potential application as a therapeutic target for EBV-associated epithelial cancers that are resistant to anti-VEGF therapy 12. It is well known that the tumour microenvironment plays an important role in controlling tumour angiogenesis 37. However, the impact of the tumour microenvironment on VM, especially in EBV-associated epithelial cancer cells, is poorly understood. A recent study revealed that cancer-associated fibroblasts regulate VM formation in hepatocellular carcinoma 38, indicating that tumour microenvironment components may play an important role in VM development in EBV-associated epithelial cancers. In this study, we further demonstrate that EBV-associated epithelial cancer cells educate the myeloid compartment via secretion and define a new regulatory role for EBV in epithelial cancer cells beyond the intrinsic control of VM.

We reasoned that M2c-like macrophages may be an important component of the tumour microenvironment in regulating VM in EBV-associated epithelial cancers, based on the classical morphological characteristics and different cell markers of TAM surrounding the VM. Consistently, the DEGs of EBV-infected epithelial cancer cells were rich in macrophage modulatory factors compared to their EBV-negative counterparts and after EBV genome destruction using the CRISPR-Cas9 system. This virus plays an important role in promoting the polarization of M2 TAM. Previous studies have indicated that hepatitis B virus (HBV) 39, human papilloma virus (HPV) 40, swine influenza virus 41, and rhinovirus 42 are involved in the regulation of M2 TAMs. It has also been found that M2 TAM infiltration accounts for 75% of NPC tissues and is associated with poor prognosis 43. However, the polarising effect of EBV on M2 TAMs in NPC remains controversial. In clinical specimens from the Netherlands, M2 TAMs have been reported to be more invasive in EBV-negative NPC than in EBV-positive NPC 44. In contrast, Su 45 and Li 46 stained NPC tissues from southern China with the EBV infection marker and the M2 marker, indicating that the positive expression rate of M2 TAMs is highly correlated with EBV infection, which is consistent with our findings. This discrepancy may be due to the regional differences in the proportions of keratinising/non-keratinising patients with NPC, where 77 (86%) of the 91 patients with NPC had keratinisation carcinoma in the Netherlands, but more than 95% of the patients in southern China were diagnosed with non-keratinising carcinoma, including those in our study population. Although the results of Su 45 and Li 46 studies were consistent with our results, a different M2 subtype with other cytokine profiles probably existed in our EBV-associated NPC. In addition to the increased level of CD206 positive M2 subtypes in EBV-associated NPC, we found that the predominant macrophage subtypes were CD163-positive M2 subtypes macrophages, which can be defined as M2c macrophage 21, 22 based on evidence of combination CSF-1 stimulation, not only in EBV-associated NPC but also in EBVaGC. To date, only a few studies have investigated the relationship between M2c macrophage upregulation and EBV infection. As a relatively specific marker for M2 macrophages, CD163 has been reported to be closely correlated with some EBV-associated tumours, such as classical Hodgkin's lymphoma 47, diffuse large B-cell lymphoma 48 and NPC 49. EBER-challenged NPC cell lines have been reported to polarise macrophages towards a tumour-promoting phenotype, perhaps through CSF-1 50. However, the biological mechanisms underlying the relationship between the upregulation of M2c macrophages and EBV infection remain unclear. In this study, we revealed a clear biological mechanism for M2c macrophage upregulation in EBV-associated epithelial cancer. Upregulation of CSF-1 in EBV-associated epithelial cancer is not only important for monocyte migration, but can also drive the differentiation of monocytes to M2c phenotypes. Our previous results 12 suggested that VM should be considered as an anti-angiogenic therapy target in patients with EBV-associated epithelial cancers. Combination therapy with inhibitors targeting VEGF receptor and intrinsic molecular pathways (HIF-1α) that regulate EBV-induced VM formation provides a means to circumvent the development of resistance to conventional anti-angiogenesis therapy. In this study, we found another key cellular component of the tumour microenvironment that is extensively involved in VM formation is represented by M2c macrophage. The inhibitors targeting HIF-1α also can inactivate the tumour microenvironment-derived extrinsic signals (CSF1 and CCL5) to further improve the response of targeting intrinsic molecular pathways to overcome the resistance to anti-VEGF therapy.

VM is a marker of poor prognosis in patients with malignant tumours 51, 52 and presents a challenge for anti-angiogenic treatments targeting ECs 53, 54. To date, there is no evidence that VM is a biomarker for predicting the clinical efficacy of anti-angiogenic therapy in cancer 55. In this study, although VM scores positively correlated with M2c macrophages, M2c macrophages could not be used as biomarkers to predict the efficacy of anti-angiogenic agent therapy. This is primarily because VM per se does not respond to anti-VEGF therapy. VM forming cells are cancer cells, not ECs, and therefore VM is not abated by antiangiogenic treatments, but rather induced by them. Although we reported that VM performed well in predicting the therapeutic efficacy of anti-angiogenic agents in patients with EBV-associated epithelial cancer, there is still a limitation that deserves further discussion. As a secondary analysis of retrospective data, it is challenging to control for various potential confounding factors among the participants. Therefore, it is necessary to design comprehensive prospective multicentre studies to verify the prediction of VM in the effectiveness of anti-angiogenic agent.

In summary, our work defines a secretory crosstalk between tumour cells and the immune microenvironment in EBV-associated epithelial cancer, highlighting an unexpected role of EBV in epithelial cancer cells controlling VM formation via M2c-like macrophages.

## Supplementary Material

Supplementary figures.
