# Supplementary material for: EBV-associated epithelial cancers cells promote vasculogenic mimicry formation via a secretory cross-talk with the immune microenvironment
Source: Theranostics. 2024 Aug 19;14(13):5123–40. doi: 10.7150/thno.100171 (PMC11388080; doi:10.7150/thno.100171)
Supplement: Supplementary file 1 — Supplementary figures. [file thnov14p5123s1.pdf]

EBV-associated epithelial cancers cells promote vasculogenic mimicry formation via a secretory cross-talk with the immune microenvironment

Tong Xiang, Fengze Sun, Tingting Liu, Jingjing Zhao, Jieying Yang, Dijun Ouyang, Hao Chen, Qijing Wang, Yongqiang Li, Jia He Chaopin Yang, Xinyi Yang, Yuanyuan Chen, Yan Tang, Desheng Weng, Qiuzhong Pan, Qi Yang, Jianchuan Xia

Supplementary figures

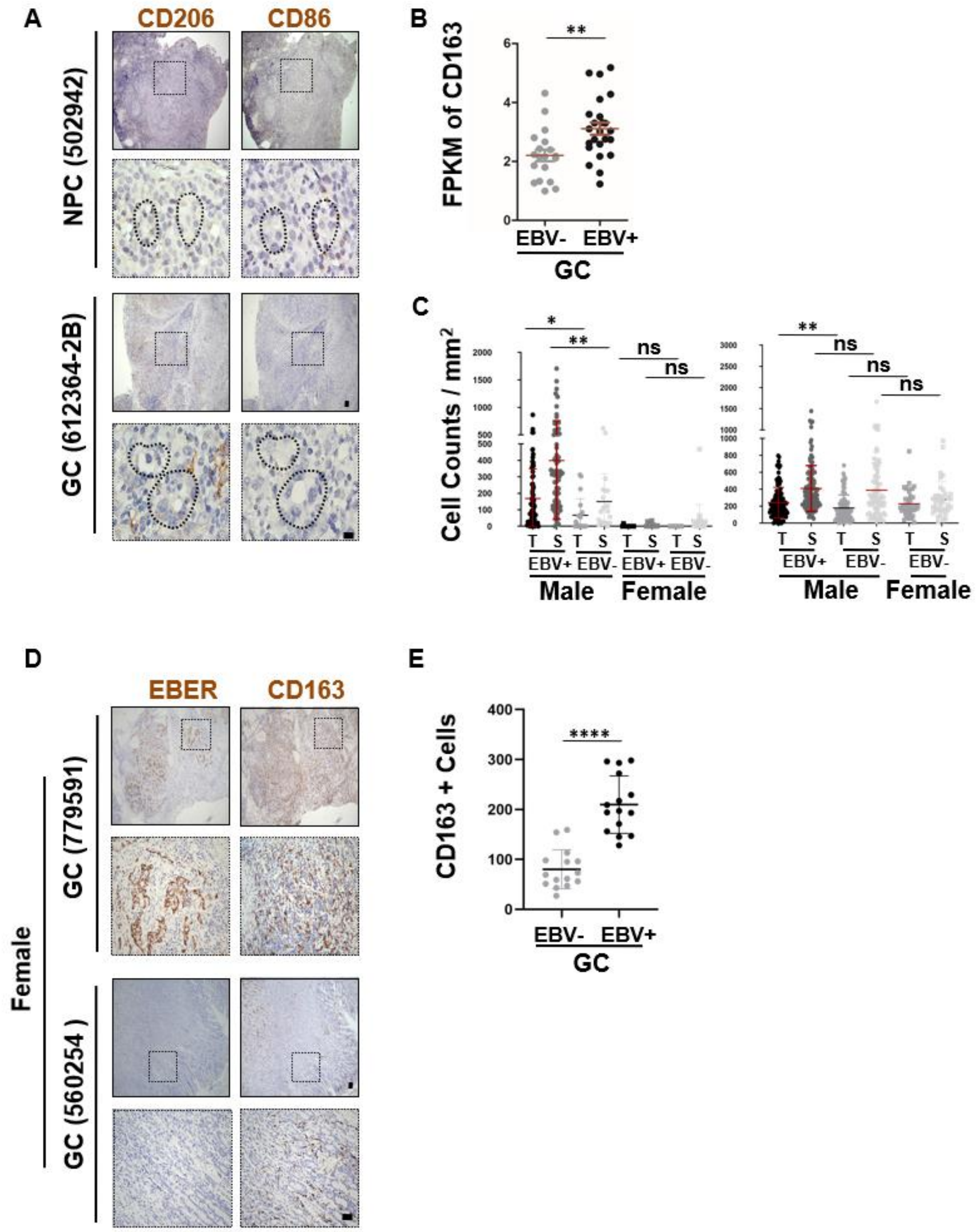

Fig. S1 The Markers of macrophages surrounded the VM. **A.** (top) NPC and (bottom) EBV-positive gastric carcinoma (GC) serial sections were stained with antibodies targeting human CD206 and CD86. Short

black scale bars = 100 μm, long black scale bars = 10 μm. **B.** The FPKM values of the CD163 in 19 EBV-negative and 24 EBV-positive gastric carcinoma cases by transcriptome sequencing. Mean ± SD, two-tailed unpaired t-test. **p < 0.01. **C.** IHC scores of the indicated macrophage cells in two EBV-negative and six EBV-positive male NPC biopsies (left) or in six EBV- negative and ten EBV- positive gastric carcinoma biopsies (right). Means ± SD, two-tailed Mann–Whitney test. ns: not significant, *p < 0.05, **p < 0.01. **D.** EBV-positive and -negative female gastric carcinoma (GC) serial sections were stained with antibodies targeting human EBER and CD163. Short black scale bars = 100 μm, long black scale bars = 10 μm. **E.** Number of CD163 positive cells counted as in D. Mean ± SD, n=5, two-tailed unpaired t-test, ****p < 0.0001.

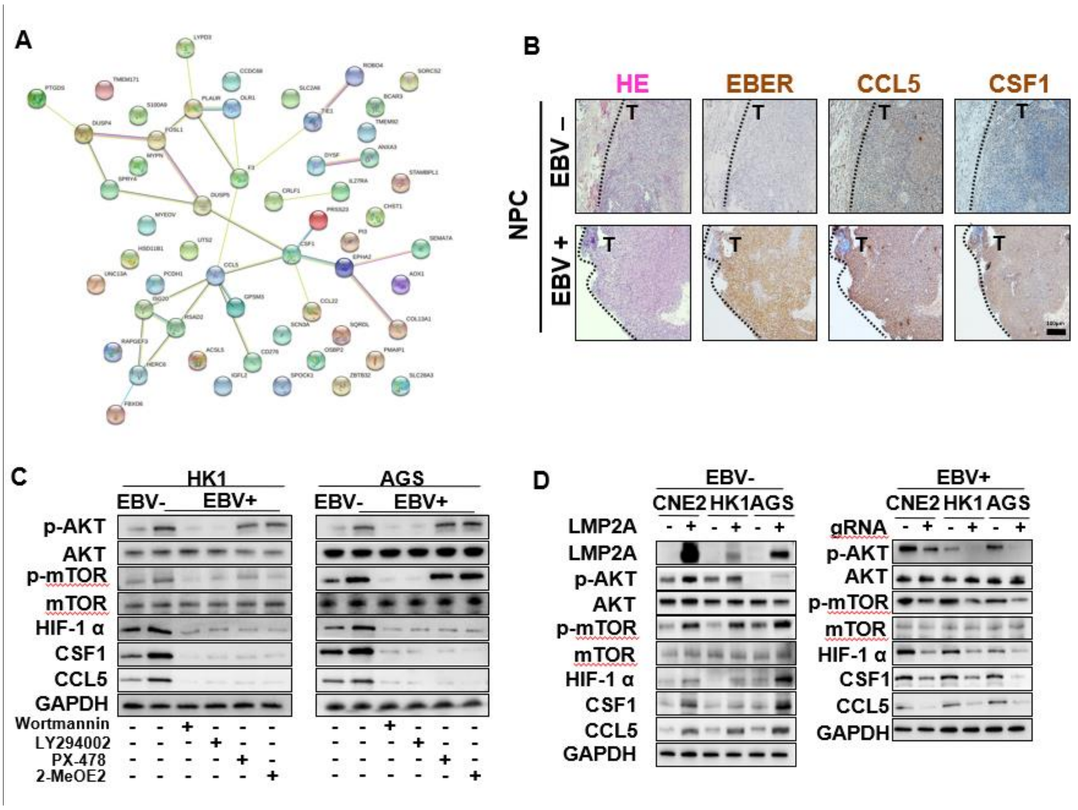

Fig. S2 EBV-infected epithelial cancer cells favour secretion of macrophage modulatory factors via the AKT/mTOR/HIF-1α pathway. **A.** Top 10 candidate core genes from the PPI network of the DEGs. **B.** Serial sections of EBV-negative and EBV-positive NPC tissues were stained with H&E, EBER and antibodies targeting CCL5 and CSF1. Scale bars = 500 μm. **C.** CNE2, HNE1, and AGS cells were transfected with empty vector or construct encoding LMP2A. Activation of the AKT/mTOR/HIF-1α signalling pathway and the levels of two macrophage modulatory factors (CSF1 and CCL5) were determined by immunoblotting. **D.** Immunoblots of the AKT/mTOR/HIF-1α signalling pathway and two macrophage modulatory factors (CSF1 and CCL5) of EBV- and EBV+ HK1 or AGS cells following LY294002 (50 μM), Wortmannin (1 μM), PX-478 (1 μM), and 2MeOE2 (1 μM) treatment for 12 h.

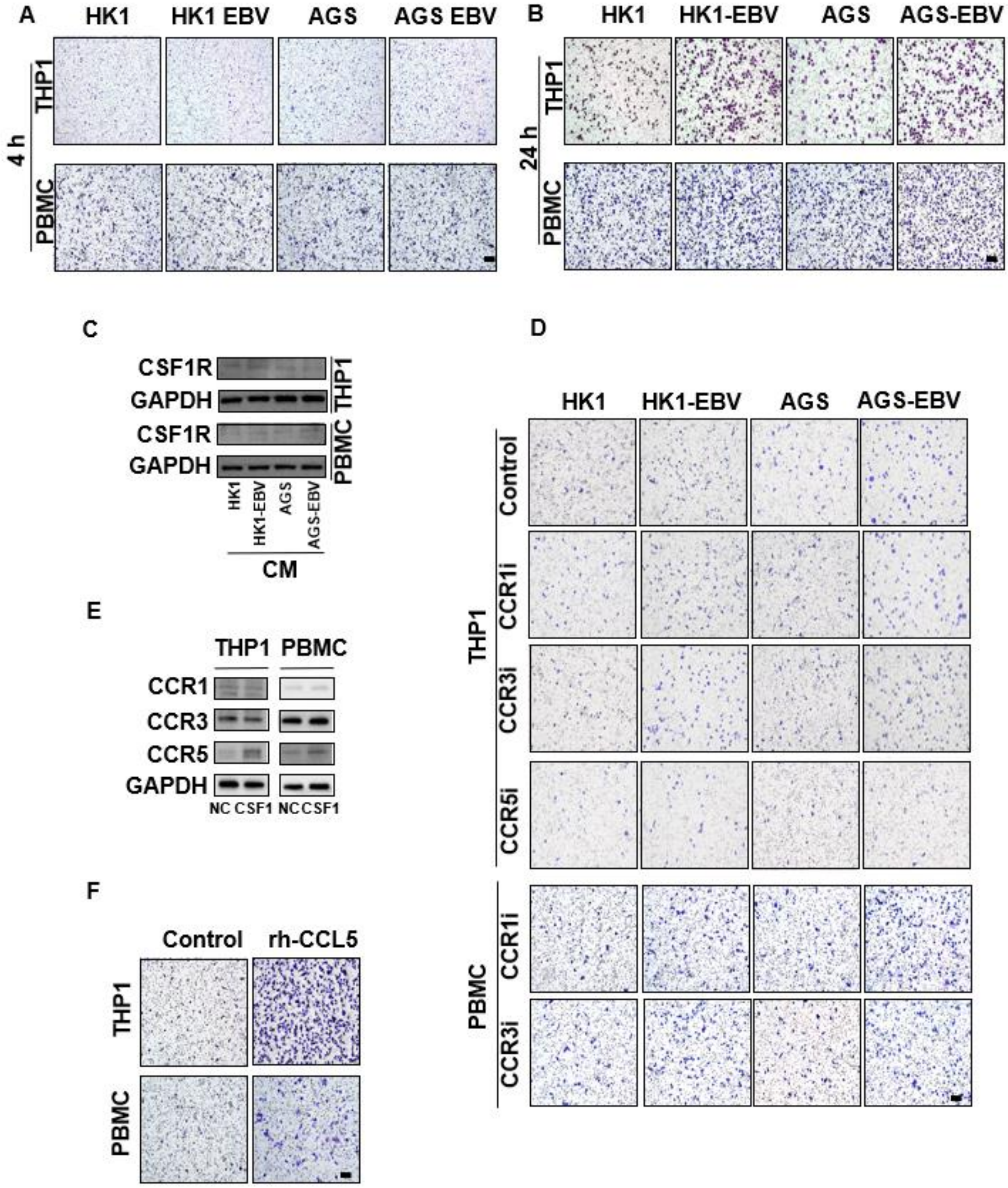

Fig. S3 EBV-associated epithelial cancer cells increase monocyte migration. A. Images of monocyte migration induced by CM of EBV-infected epithelial cancer cells and their parental cells. Images were taken 4 h after seeding on chamber. Scale bars = 100 μm. **B.** Images of monocyte

migration induced by CM of EBV-infected epithelial cancer cells and their parental cells. Images were taken 24 h after seeding on chamber. Scale bars = 100 μm. **C.** CSF1R levels on the THP1 and PBMC-derived monocyte stimulated by EBV-positive tumour cells CM after 24 h. **D.** Images of monocyte migration induced by CM of EBV-infected epithelial cancer cells and their parental cells in the presence of CCR1, CCR3, and CCR5 inhibitor and rh-CCL5. Images were taken 4 h after seeding on chamber. Scale bars = 100 μm. **E.** Levels of CCR1, CCR3, and CCR5 on the THP1 and PBMC-derived monocyte stimulated by CSF1 after 24 h. **F.** Images of monocyte migration induced by recombinant human CCL5 (rh-CCL5). Scale bars = 100 μm.

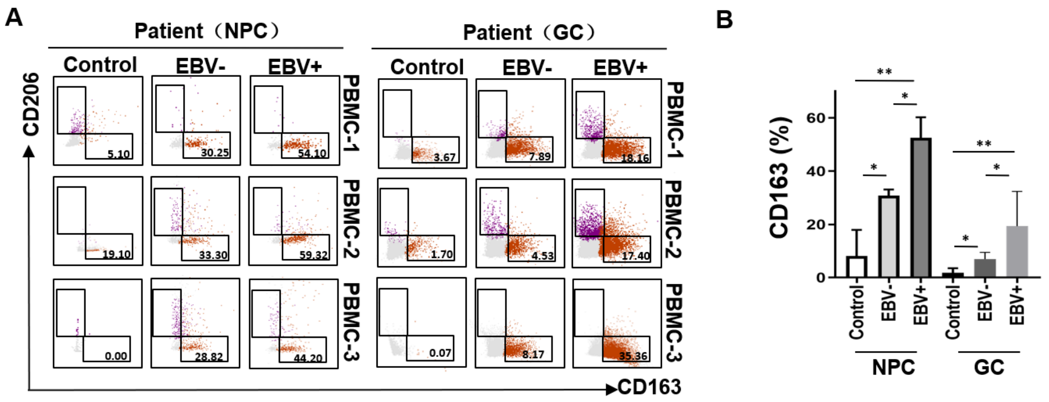

Fig. S4 EBV-associated epithelial cancer cells induce M2c-like macrophages differentiation. A. Flow cytometry dot plots from PBMC-derived monocytes of three donor showing CD163 and CD206 levels after treatment with CM of patient derived EBV-infected and EBV-uninfected epithelial cancer cells. **B.** %CD163 levels after treatment with CM of

patient derived EBV-infected and EBV-uninfected epithelial cancer cells. Mean ± SD, two-tailed unpaired t-test. *$p < 0.05$, **$p < 0.01$.

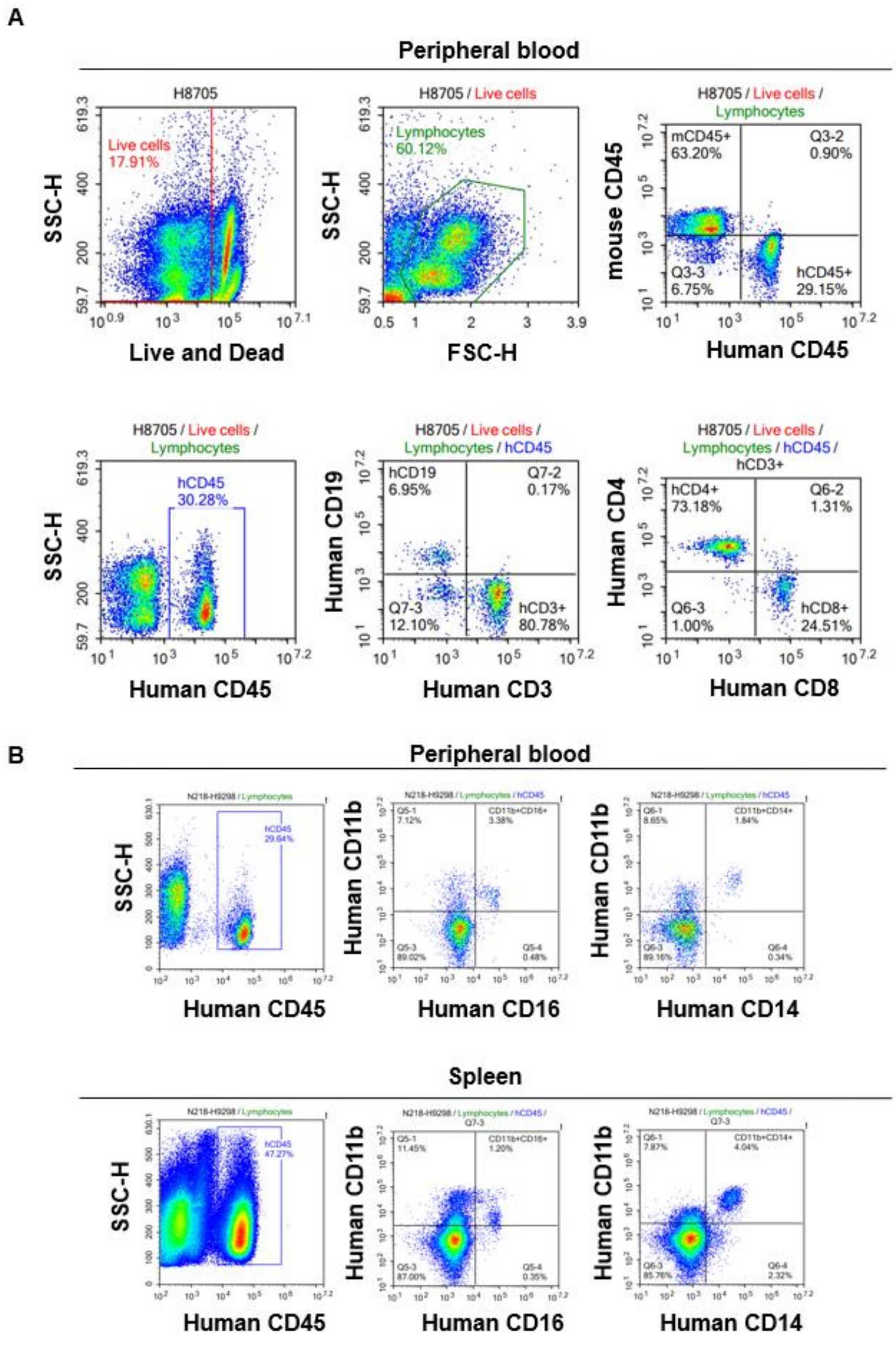

Fig. S5 Humanised HLA-A24 transgenic NSG mice. A. Flow cytometry dot plots from peripheral blood of one humanised HLA-A24 transgenic NSG mice showing CD45, CD3, CD19, CD4, and CD8 levels. **B.** Flow

cytometry dot plots from peripheral blood and spleen of one humanised HLA-A24 transgenic NSG mouse showing CD45, CD16, CD14, and CD11b levels.
